# Photomotion of Hydrogels with Covalently Attached Azo Dye Moieties—Thermoresponsive and Non-Thermoresponsive Gels

**DOI:** 10.3390/gels8090541

**Published:** 2022-08-28

**Authors:** Thorben G. Jaik, Assegid M. Flatae, Navid Soltani, Philipp Reuschel, Mario Agio, Emiliano Descrovi, Ulrich Jonas

**Affiliations:** 1 Department Chemistry—Biology, University of Siegen, Adolf-Reichwein-Strasse 2, 57076 Siegen, Germany; thorben.jaik@uni-siegen.de; 2 Department Physics, University of Siegen, Walter-Flex-Str. 3, 57072 Siegen, Germany; assegid.flatae@uni-siegen.de (A.M.F.); navid.soltani@uni-siegen.de (N.S.); philipp.reuschel@gmail.com (P.R.); mario.agio@uni-siegen.de (M.A.); 3 National Institute of Optics (INO), National Research Council (CNR), Via Nello Carrara 1, 50019 Sesto Fiorentino, Italy; 4 Department of Applied Science of Technology (DISAT), Politecnico di Torino, Corso Duca degli Abruzzi 24, 10129 Torino, Italy; emiliano.descrovi@polito.it

**Keywords:** photoactuation, azo hydrogels, LCST

## Abstract

The unique photomotion of azo materials under irradiation has been in the focus of research for decades and has been expanded to different classes of solids such as polymeric glasses, liquid crystalline materials, and elastomers. In this communication, azo dye-containing gels are obtained by photocrosslinking of non-thermoresponsive and lower critical solution temperature type thermoresponsive copolymers. These are analysed with light microscopy regarding their actuation behaviour under laser irradiation. The influences of the cloud-point temperature and of the laser power are investigated in a series of comparative experiments. The thermoresponsive hydrogels show more intense photoactuation when the cloud-point temperature of the non-crosslinked polymer is above, but closer to, room temperature, while higher laser powers lead to stronger motion, indicating a photothermal mechanism. In non-thermoresponsive gels, considerably weaker photoactuation occurs, signifying a secondary mechanism that is a direct consequence of the optical field-azo dye interaction.

## 1. Introduction

Photoactuated polymer-based hydrogels are water-swollen networks with macromolecular chain segments that experience mechanical deformation under light irradiation. The photoactuation of these systems can be traced to essentially two mechanisms: A photothermal mechanism, in which, e.g., embedded nanoscaled materials serving as absorbers are heated by light and act on either elastomers or thermoresponsive polymers [1,2,3], or a photochemical mechanism, in which dyes such as spiropyranes, hexaarylbiimidazoles, and azobenzenes lead to deformation upon light-induced changes in their molecular structure [4,5].

Photothermal effects can be observed in azo dye-containing systems as well. Under irradiation with light of appropriate wavelength according to the dye absorption and high intensity (100–400 mW cm^−2^), the temperature of films of polymer-dye blends [6], azopolymers [7], and liquid-crystalline elastomers containing azobenzene moieties [8] can increase by up to 80 K temperature difference. However, the temperature rise depends specifically on the characteristics of the system, as an increase of only a few Kelvins has been observed in glassy polymers [9] and thick films of nematic liquid-crystalline elastomers [10].

An application of azobenzene-containing copolymers was reported for artificial extracellular matrices (ECM) that can be switched in stiffness and swelling states by irradiation with light [11]. In these examples, lower critical solution temperature (LCST)-type thermoresponsive polymers in solution and hydrogels containing (hydroxy-) azobenzene units were alternatingly irradiated with UV- and visible light to switch between the *trans-* and *cis-*state of the dye. In the *trans-*state, the systems are more hydrophobic compared to the *cis*-state, and thus, lowered in their cloud point and swelling ratio. Consequently, both the cloud point of the polymers in solution, as well as the swelling ratio of the corresponding gels can be switched by irradiation with light. It must be noted that the thermal relaxation from the *cis*- to the *trans-*isomer is slow in the specific azobenzene derivatives used.

A widely explored photoresponsive gel system is the combination of cyclodextrin and azobenzene-containing compounds or polymers. In these systems, the azobenzene derivatives can be complexated by cyclodextrin in the *trans*-state but not in the *cis*-state. Therefore, the dye and cyclodextrin form crosslinks that are reversible under irradiation with light of suitable wavelength. As a consequence, it is possible to switch between the sol- and the gel-state [12,13,14,15,16,17,18,19,20,21,22,23,24,25,26].

In the present study, we aim to expand the range of photoactive soft materials containing azo dyes. For this purpose, we synthesised photocrosslinkable, hydrophilic copolymers by free radical polymerisation with *o*-methyl red attached to the backbone as push-pull azo dye. By photocrosslinking and swelling with water, azo dye-containing hydrogels are obtained. Two classes of gels were prepared for which photoactuation was investigated. The first one is a non-thermoresponsive gel based on *N*-hydroxyethyl acrylamide (HEAm). The second system comprises a hydrogel with LCST-behaviour, prepared from *N*-isopropylacrylamide (NiPAAm). The obtained hydrogels were irradiated with a focussed laser and photoactuation was tracked by light microscopy. A comparison of the systems allows researchers to separate photochemical and photothermal effects.

## 2. Results and Discussion

Thermoresponsive and non-thermoresponsive copolymers were synthesised by free radical polymerisation from the hydrophilic monomers NiPAAm and HEAm, a *N*-hydroxyethyl acrylamide ester of *o*-methyl red (oMREAm), and the photocrosslinker 4-benzophenone acrylamide (BPAAm). From these copolymers, hydrogels were obtained by photocrosslinking, and subsequent swelling in water. While NiPAAm introduces LCST-type thermoresponsiveness in aqueous copolymer solution, the cloud-point temperature may be tuned by varying the ratio of NiPAAm to HEAm, which is a highly polar monomer and, by itself, does not show thermoresponsiveness. A higher amount of HEAm in a PNiPAAm-copolymer increases the cloud-point temperature, as more hydrophilic comonomers lead to a decrease in cloud-point temperature in LCST-type copolymers. [27] HEAm was chosen as oMREAm is a pH indicator and additional factors such as protonation from a monomer such as methacrylic acid had to be avoided to reduce complexity of the systems.

**Figure 1:**
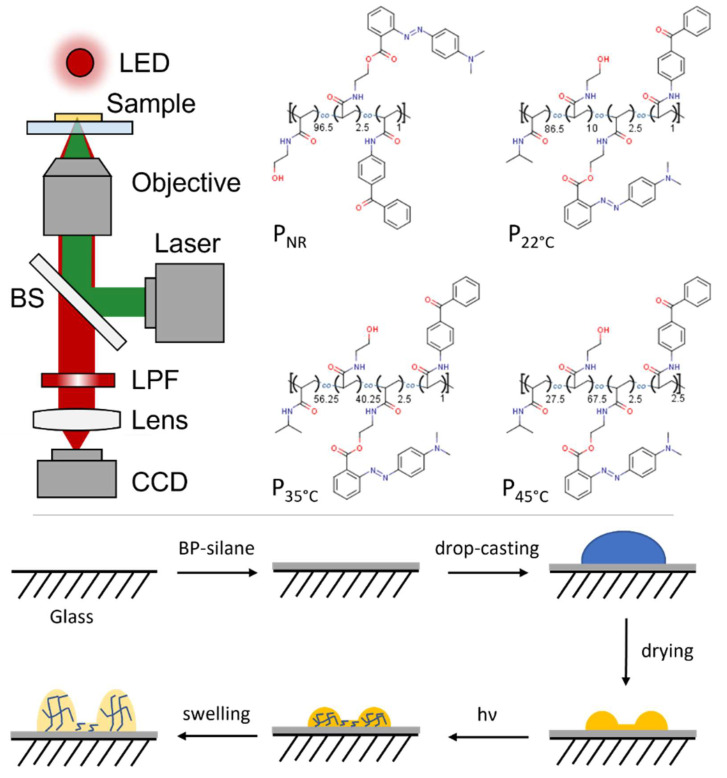
**Top left**: Sketch of the optical setup used in photoactuation experiments including laser, light-emitting diode (LED), objective, sample, dichroic beamsplitter (BS), longpass filter (LPF), lens, and charge-coupled device (CCD) camera; **Top right**: Structures and monomer feed composition of the different non-thermoresponsive (P_NR_) and thermoresponsive copolymers (P_22°C_, P_35°C_, P_45°C_) employed in this study; **Bottom**: Sample preparation process of o-methyl red-containing hydrogels for photoactuation experiments, involving functionalisation of the glass surface with a silane of the photocrosslinker benzophenone, drop-casting a solution of the respective copolymer, drying, photocrosslinking, and subsequent swelling.

Thus, four copolymers, which either do not respond to temperature or show LCST-behaviour over a range close to and above room temperature, were synthesised to investigate their photoactuation capacities in comparative optical experiments. P_NR_ does not show thermoresponsive behaviour. A 1 w% aqueous solution of P_22°C_ features a cloud-point temperature at 22 °C, P_35°C_ at 35 °C, and P_45°C_ at 45 °C (cf. Figure 1, ESI Figure S5b). A similar dye content was targeted during the polymer synthesis for these copolymers with an oMREAm monomer feed of 2.5%. The BPAAm feed was set to 1% for P_NR_, P_22°C_, and P_35°C_, but to 2.5% for P_45°C_, as photocrosslinking and attachment to a surface proved challenging at 1% BPAAm for P_45°C_ due to the increased hydrophilicity and the corresponding higher swelling ratio of this particular polymer.

The copolymers were drop-cast on benzophenone-functionalised glass surfaces and subsequently photocrosslinked with UV-light (302 nm, 28.4 J cm^−2^). The glass slides were treated prior with 4-(3-triethoxysilyl) propoxybenzophenone to allow for covalent attachment of the polymer network to the glass.

The films were swollen in water before they were fixed in a home-built optical setup. The sample is illuminated from the bottom with a focussed green laser (532 nm wavelength) and from the top with a red LED in a widefield configuration. Scattered light from the sample is collected via the focussing objective (50×, 0.95 NA) and the green light is spectrally blocked via a dichroic beamsplitter and a longpass filter. A lens focusses the scattered light onto a high-resolution charge-coupled device (CCD) camera, which monitors the sample’s mechanical response (cf. Figure 1).

Three different sets of experiments were performed:

1. The ability for photoactuation was compared between a gel of the non-thermoresponsive P_NR_ and a gel of the thermoresponsive P_22°C_. The LCST-behaviour of P_22°C_ was then turned off by changing the liquid medium to isopropanol, to study the effect of thermoresponsiveness on photoactuation.
2. Hydrogels of copolymers with different cloud points were photoactuated at high laser powers (in the range of 3.75 mW) to further elucidate photothermal effects.
3. A gel of P_22°C_ was illuminated at different laser powers (from 85 µW to 3.75 mW) to correlate the degree of photoactuation with the light intensity.

A common prerequisite for all experiments was a minimal thickness of the gels (several tens of µm) to allow significant mechanical deformation that is observable in optical microscopy. In order to achieve this thickness, the gels were prepared by drop-casting from water-methanol mixtures on hydrophobic surfaces instead of spin-coating. This procedure leads to a coffee-stain effect, which results in a thicker polymer layer at the edge of the initial droplet. The thicker part of the gel layer (between 25 and 50 µm in the swollen state with swelling ratios of about 2.5) was probed in the photoactuation experiments. Defect structures in the hydrogel films were exploited to follow photoactuation in the optical microscope. These imperfections are usually folds of hydrogel that are formed at high swelling ratios, where the gel partially detaches from the surface. The wavelength of the laser (532 nm) was chosen to match the absorption band of the employed azo dye (cf. ESI Figure S5a).

### 2.1. Effect of Thermoresponsiveness

When irradiating the non-thermoresponsive gel of P_NR_, the only interaction of light with the system is expected to be with the azo moieties, which undergo *trans*-*cis*-isomerisation attaining a photostationary equilibrium. Based on the reported photomotion of other azo dye-containing polymer systems, photoactuation is expected.

**Figure 2:**
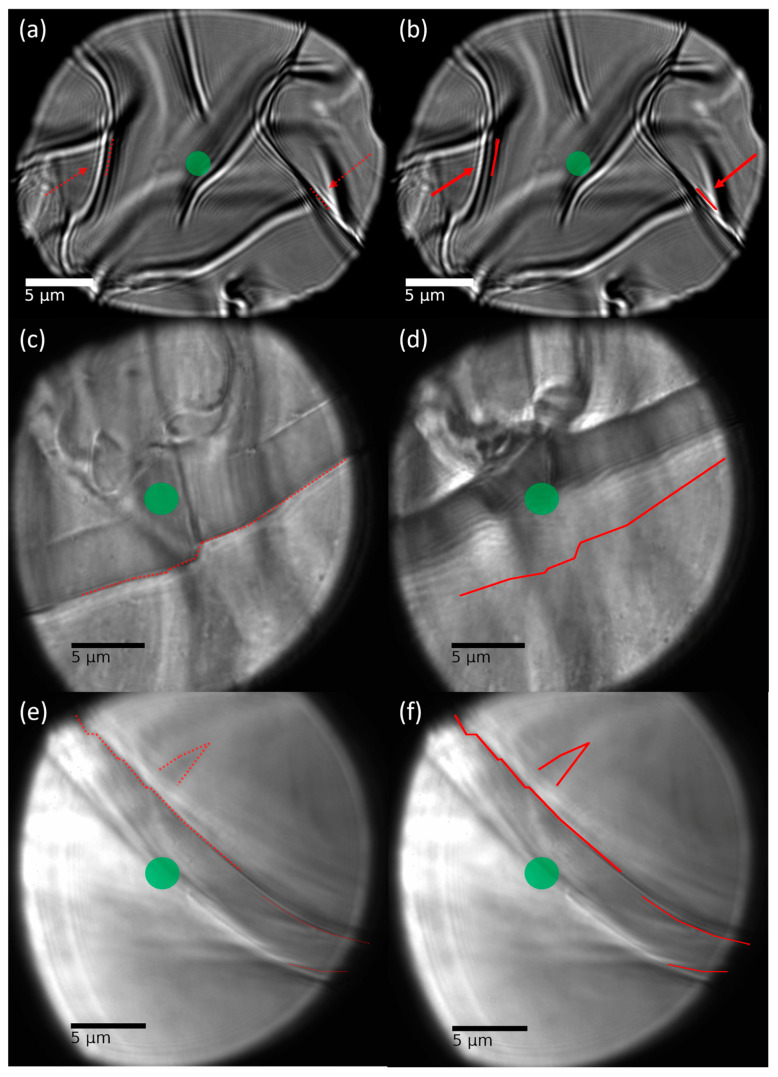
Light microscopy images of pristine (left panels) and photoactuated regions (right panels) of gel layers prepared from (**a**,**b**) poly(HEAm_96.5%_-co-o-MREAm_2.5%_-co-BPAAm_1%_) P_NR_, swollen in water, (**c**,**d**) poly(NipAAm_86.5%_-HEAm_10%_-co-o-MREAm_2.5%_-co-BPAAm_1%_) P_22°C_, swollen in water, (**e**,**f**) the same polymer in isopropanol. Photoactuation was performed with a laser at λ = 532 nm and 2600 µW (irradiation region marked by a green spot). Red lines and arrows indicate notable gel features that were photoactuated. Videos of the photomotion are provided in the download section of the supporting information.

Indeed, in the investigated hydrogel system, photoactuation is observed, but in the water-swollen gel, only minute mechanical deformation is visible in the optical microscopy images in Figure 2a,b (cf. ESI Movie S1). The motion is in the sub-µm range, but effective over areas as large as 10 µm away from the illumination centre. In Figure 2, the pristine gel without illumination on the left is compared with the illuminated sample on the right. The images of the irradiated gels were taken three to five seconds after the start of laser exposure.

To better illustrate this minute photoactuation, which is barely visible in the microscopic still pictures, short films of repeated actuation cycles are provided online as supplementary material (cf. ESI Movies S1–S3). These films show the reversibility of the process and the time scales over which they occur as well.

Very much in contrast, the hydrogel of the thermoresponsive P_22°C_ shows an impressively strong and fast response to irradiation. The photoactuation yields mechanical deformation more than 10 µm away from the laser (cf. Figure 2c,d, ESI Movie S2). Apparently, this substantial deformation is caused by a local collapse of the gel. Such a collapse behaviour is known for the NiPAAm-based, thermoresponsive polymers, which show lower critical solution transitions, when exceeding the temperature of the cloud point.

In order to validate the thermal nature of the volume transition, the liquid medium is exchanged from water to isopropanol, thus eliminating the LCST-behaviour. Consequently, in isopropanol the mechanical response of the gel is strongly diminished. Now, only a miniscule movement of features over short distances well below 1 µm are observed (cf. Figure 2e,f, ESI Movie S3), similar to the non-thermoresponsive hydrogel of P_NR_. Again, the distance from the laser spot at which motion occurs is above 10 µm.

All three samples have in common that motion occurs towards the centre of the laser spot. In the P_22°C_ hydrogel, response to the incident laser beam is immediate, but five seconds pass until the gel is fully collapsed. The system requires about the same timeframe to recover its initial conformation. In both cases, 90% of the collapse or restoring motion occur within 2 s (cf. ESI Movie S2). In the two non-thermoresponsive scenarios, the timeframes are considerably shorter. In less than two frames or 0.47 s, the photoactuation is complete and the gels return to their initial conformation as quickly (cf. ESI Movies S1–S3).

Comparing these results to previous observations of collapse and swelling behaviour of these photocrosslinked PNiPAAm hydrogel layer systems, which show similar volume transitions and kinetics upon thermal stimulation [28], it follows that thermoresponsiveness is a crucial factor in photoactuation of these azobenzene-containing gels. While non-thermoresponsive gels demonstrate a lower degree of motion upon irradiation, the effect in the thermoresponsive gel is magnitudes stronger. Apparently, a photothermal effect, in which the impinging light is converted into thermal energy within the hydrogel, presents the underlying mechanism to trigger thermoresponsiveness, resulting in photoactuation of the gel.

Generally, photothermal effects in molecules are a consequence of vibrational modes being excited by light. As azobenzenes show optically induced *trans-cis* isomerisation and radiationless decay of the *cis*-state is prominent; such dyes are prominent examples for photothermally active molecules [6,8,9,29,30]. The overlap of the absorption bands of the *trans-* and the *cis-*states occurring in push-pull-azobenzenes [31] apparently enhance these effects [6].

### 2.2. Cloud-Point Temperature Dependence

In the second set of experiments, a dependence of the photoactuation on the cloud point can be identified. A lower cloud point closer to the observation temperature (room temperature) yields a stronger response of the hydrogel towards laser stimulation.

**Figure 3:**
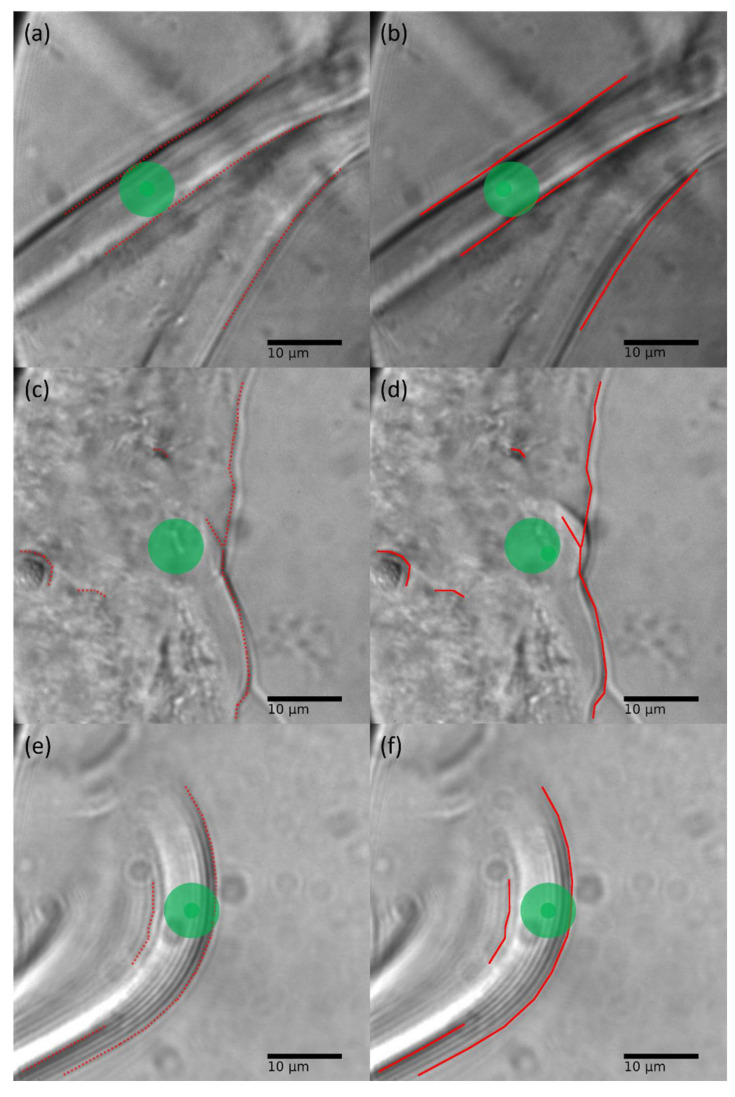
Light microscopy images of pristine (left panels) and photoactuated regions (right panels) of hydrogel layers prepared from (**a**,**b**) poly(NipAAm_86.5%_-HEAm_10%_-co-o-MREAm_2.5%_-co-BPAAm_1%_) P_22°C_, (**c**,**d**) poly(NipAAm_56.25%_-HEAm_40.25%_-co-o-MREAm_2.5%_-co-BPAAm_1%_) P_35°C_, and (**e**,**f**) poly(NipAAm_27.5%_-HEAm_67.5%_-co-o-MREAm_2.5%_-co-BPAAm_2.5%_) P_45°C_. Photoactuation was performed with a laser at λ = 532 nm and 3750 µW (irradiation region marked by a green spot). Red lines indicate notable gel features that were photoactuated.

As discussed above, the low cloud-point temperature copolymer P_22°C_ collapses locally in a matter of seconds when irradiated, pulling material over a large area towards the collapsed region (cf. Figure 3a,b). The hydrogels with medium and higher cloud-point temperature from P_35°C_ and P_45°C_ do not exhibit such intense photomotion, but only show a weakened movement towards the laser spot (cf. Figure 3c–f).

It should be noted that the photomotion in these experiments is not fully reversible and permanent deformations in the morphology of the gels remain, in particular, close to the laser spot. This can be visualised by subtracting one picture from the other, revealing changes either in the pristine gel to the irradiated gel (photoactuation) or in the pristine gel to the gel after exposure (irreversible change) (cf. ESI Figures S6, S7, S9 and S10). The nature of the irreversible change is ambiguous. In P_35°C_ and P_45°C_, the dimensions after irradiation are larger than prior to contracting it via irradiation, meaning that a higher degree of swelling is attained (cf. ESI Figures S9 and S10). In P_22°C_, on the other hand, the gel is less swollen after irradiation (cf. ESI Figure S7).

Another result of repeated or prolonged exposure to laser light is a diminished response to irradiation. With a higher number of irradiation cycles, the collapse of the gel becomes less pronounced (cf. ESI Figure S8).

To understand the nature of the irreversible changes, further research would be required. Several possible explanations are perceivable, such as photodissociation of the azo dye, photoionization, or breaking of crosslinks, which would all lead to an increased degree of swelling. However, formation of new crosslinks would decrease swelling. Based on the limited experimental evidence from light microscopy, the role of each of these different options cannot be assigned in more details.

The conclusion from this set of experiments is that the local temperature increase is obviously limited and, consequently, the photothermal effect, leading to a local collapse of the P_22°C_ gel, is not intense enough to increase the temperature to a point where also the P_35°C_ or the P_45°C_ gel collapse under otherwise identical conditions.

### 2.3. Power Dependence

In the last set of experiments, the hydrogel of P_22°C_ was exposed to laser light at different power levels. The hydrogel showed small responses to the incident laser even at low powers of around 400 µW, but considerable motion and collapse of the gel occurred only above a power of 1.2 mW, increasing with higher powers (cf. ESI Figure S6). The magnitude of the photoactuation, discussed above in Section 1, was induced at high power levels (above 2.6 mW) for this gel.

An increase in photoactuation with increasing laser power is expected when considering photothermal effects as the driving force behind the collapse and the concurrent motion of the gel. As is typical for LCST-type thermoresponsive polymers, a certain temperature must be exceeded for the phase transition to occur. Consequently, under irradiation, the local temperature has to increase past this cloud-point temperature to induce the collapse. Assuming the photothermal effect scales proportionally with the energy input, the number of collapsing network segments per volume element increases with the energy input as well, resulting in enhanced photoactuation.

The thermoreponsive gels of P_35°C_ and P_45°C_ have higher cloud-point transition temperatures, thus irradiation leads to smaller motion.

### 2.4. Reversibility of Photomotion:

The reversibility of photoactuation is linked to the network structure of the swollen gel. In thermoresponsive hydrogels, a laser-induced local collapse results in a strain acting on the circumjacent material. In this situation, two factors contribute to the restoring force: (1) The swollen gel in the surroundings of the collapse shows rubber elasticity [32] and exerts a restoring force of entropic origin on the deformed parts. (2) When laser irradiation is stopped, the local heat dissipates, allowing the temperature in the irradiation spot to drop back below the cloud-point temperature. At this point, the gel reswells and releases the strain.

In photomotion involving a non-thermoresponsive system, the deformation force results from chromophore alignment in the irradiation spot. The strain generated by the stretched polymer network ensures restoring the original shape of the gel after the optical stimulus is removed.

### 2.5. Photoactuation Models:

**Scheme 1:**
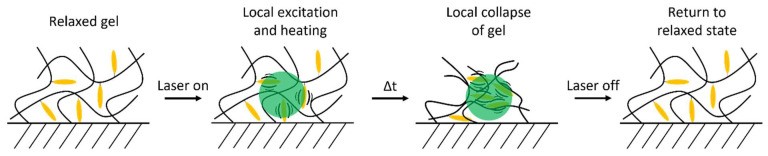
Cartoon of the photoactuation process of thermoresponsive o-methyl red-containing hydrogels. Local excitation of dye side chains by laser irradiation induces heating, which leads to a local collapse and deformation of the gel. The gel returns to its original conformation when the laser is shut off and the heat is dissipated to the environment.

From the three sets of experiments, two different pathways of photoactuation can be postulated in these *o*-methyl red-containing hydrogels. The first pathway involves an LCST-type thermoresponse triggered by photothermal effects, induced by repeated *cis-trans* photoisomerisations of the azo dye molecules under irradiation with a focussed laser beam. This pathway is summarised in Scheme 1.

The second pathway is more intricate, as in the absence of thermoresponsive behaviour, photomotion towards the irradiated area still occurs, although smaller in scale. This is the case in the non-thermoresponsive gels of P_NR_ and of P_22°C_ in isopropanol. However, if photo-induced heating was the underlying cause for photoactuation by volume expansion in these systems, the gels should swell slightly, resulting in a motion away from the incident beam.

In the photoactuation experiments for non-thermoresponsive systems, the opposite motion towards the illuminated region is observed. Apparently, additional mechanisms of photoactuation must be considered to explain these experimental findings. Such an explanation may be drawn from the models that attempt to explain the formation of surface relief gratings (SRGs), which are introduced below.

SRG formation, which has been described for glassy, amorphous, and liquid crystalline polymers and materials, usually requires high concentrations of azo dyes. Under these conditions, the dyes are in proximity to each other and can perform collective motions. In the polymer systems here, the degree of substitution along the backbone is low (<2.5 mol%) and the volume fraction in the swollen gel is even lower, because of dilution with the swelling solvent. In contrast, the mobility of the chromophores is considerably higher in these visco-elastic solids compared to the more rigid matrixes in SRG-formation.

Under these conditions, only a few reported models may be suitable as reference. These are the orientation model [33,34,35], the gradient force model [8,36,37,38,39,40], and the mean field model [41,42]. These models have in common that they assume reorientation of the chromophore during irradiation in the material by *trans-cis-trans* isomerisation cycles and alignment of the chromophores in the optically induced electric field. This reported mechanism may also serve as explanation for the here observed photomotion in the gels. Forced alignment of the chromophores by dipolar interactions in the irradiation field would lead to contraction of the gel, as the chromophores pull the attached segments of the network along with them. However, this motion is limited, as only a comparably weak force can be generated by a single chromophore.

## 3. Conclusions

The above results demonstrate that the combination of thermoresponsive, water swollen polymer networks with the unique photoresponse of azo dyes provide novel materials with extended photomotion, which may be particularly applicable in the context of living organisms owing to the aqueous medium of the hydrogels. Potentially, such systems could be used for mechanical stimulation of cells with the remote, non-invasive trigger light, or as optically driven actuators for soft robotics in biological environments. For this future field of application, tests in cell cultures to investigate biocompatibility are a necessary next step. In such environments, also thin films, structured materials, as well as macroscopic gels need to be investigated.

## Data Availability

All data is available either in this article or in the Supplementary Materials.

## Supplementary Materials

The following supporting information can be downloaded at: https://www.mdpi.com/2310-2861/8/9/541/s1, Table S1: Feed composition in mol% and reaction parameters for the different free radical copolymersations.; Table S2: Copolymer characteristics as determined by UV-vis spectroscopy and GPC. The built-in ratios for o-MREAm and BPAAm are given in weight percentages and the percentage of the monomer built-in compared to the feed composition. The HEAm:NiPAAm ratio was determined by ^1^H NMR spectroscopy.; Figure S1: ^1^H NMR spectrum (500 MHz) in MeOD of a poly(HEAm-co-o-MREAm-co-BPAAm) copolymer (P_NR_) (feed: HEAm:o-MREAm:BPAAm 96.5:2.5:1).; Figure S2:^1^H NMR spectrum (400 MHz) in MeOD of a poly(NiPAAm-co-HEAm-co-o-MREAm-co-BPAAm) copolymer (P_22°C_) (feed: NiPAAm:HEAm:o-MREAm:BPAAm 87.2:9.2:2.6:1).; Figure S3:^1^H NMR spectrum (500 MHz) in D2O of a poly(NiPAAm-co-HEAm-co-o-MREAm-co-BPAAm) copolymer (P_35°C_) (feed: NiPAAm:HEAm:o-MREAm:BPAAm 55.9:40.4:2.7:1).; Figure S4:^1^H NMR spectrum (500 MHz) in D2O of poly(NiPAAm-co-HEAm-co-o-MREAm-co-BPAAm) copolymer (P_45°C_) (feed: NiPAAm:HEAm:o-MREAm:BPAAm 27.7:67.3:2.5:2.5).; Figure S5: (a) UV-vis spectrum of a photocrosslinked film of poly(HEAm_96.5%_-co-o-MREAm_2.5%_-co-BPAAm_1%_) P_NR_ swollen in water at 26 °C, with the wavelength of the green laser used in this marked with a green vertical line at 532 nm. b) Turbidity measurements at 780 nm of aqueous 1 w% solutions of poly(NipAAm_86.5%_-HEAm_10%_-co-o-MREAm_2.5%_-co-BPAAm_1%_) P_22°C_, poly(NipAAm_56.25%_-HEAm_40.25%_-co-o-MREAm_2.5%_-co-BPAAm_1%_) P_35°C_, and poly(NipAAm_27.5%_-HEAm_67.5%_-co-o-MREAm_2.5%_-co-BPAAm_2.5%_) P_45°C_. On the right: structures of the copolymers used in this study with the feed ratios of monomers in the synthesis.; Figure S6:A series of light microscopy images of a hydrogel of poly(NipAAm_86.5%_-HEAm_10%_-co-o-MREAm_2.5%_-co-BPAAm_1%_) P_22°C_ showing left: non-irradiated gel with accumulative irreversible change of irradiations at previous stages of power; middle: difference pictures between the non-irradiated and the irradiated gel with inverted colour scheme (laser at λ = 532 nm); right: irradiated gel after 3 s at (a) 85 µW, (b) 376 µW, (c) 1210 µW, and (d) 3750 µW. The green shaded area shows the approximate dimensions of the laser spot.; Figure S7: Light microscopy images of a hydrogel of poly(NipAAm_86.5%_-HEAm_10%_-co-o-MREAm_2.5%_-co-BPAAm_1%_) P_22°C_ showing (a) the pristine hydrogel, (b) the hydrogel when irradiated with a laser (λ = 532 nm, 3750 µW), (c) after 3 s of exposure, and (d) after 60 s of exposure; Figure S8: Light microscopy images of a hydrogel of poly(NipAAm_86.5%_-HEAm_10%_-co-o-MREAm_2.5%_-co-BPAAm_1%_) P_22°C_ irradiated with laser (λ = 532 nm) at 3750 µW (a) for the first time, (b) after cycling five times between 3 s irradiation intervals and relaxation, (c) after cycling twenty times between 3 s irradiation intervals and relaxation. The red lines in (b) and (c) show the positions of the notable dark features in (a); Figure S9: Light microscopy images of a hydrogel of poly(NipAAm_56.25%_-HEAm_40.25%_-co-o-MREAm_2.5%_-co-BPAAm_1%_) P_35°C_ showing (a) left to right: the pristine gel, a difference picture between the non-irradiated and the irradiated gel, the irradiated gel (laser at λ = 532 nm, 3750 µW), and (b) left to right: the pristine gel, a difference picture between the non-irradiated gel before and after exposure to laser light, the gel after exposure to laser light. The green shaded area shows the approximate dimensions of the laser spot. The difference pictures are shown with inverted colour scheme.; Figure S10: Light microscopy images of a hydrogel of poly(NipAAm_27.5%_-HEAm_67.5%_-co-o-MREAm_2.5%_-co-BPAAm_2.5%_) P_45°C_ showing (a) left to right: the pristine gel, a difference picture between the non-irradiated and the irradiated gel, the irradiated gel (laser at λ=532 nm, 3750 µW), and (b) left to right: the pristine gel, a difference picture between the non-irradiated gel before and after exposure to laser light, the gel after exposure to laser light. The green shaded area shows the approximate dimensions of the laser spot. The difference pictures are shown with inverted colour scheme.; Video S1: MovieS1_PNR_aqueous; Video S2: MovieS2_P22C_aqueous; Video S3: MovieS3_P22C_Isopropanol [43,44,45].
