# Correction: Jaik et al. Photomotion of Hydrogels with Covalently Attached Azo Dye Moieties—Thermoresponsive and Non-Thermoresponsive Gels. *Gels* 2022, *8*, 541

**DOI:** 10.3390/gels9050411

**Published:** 2023-05-15

**Authors:** Thorben G. Jaik, Assegid M. Flatae, Navid Soltani, Philipp Reuschel, Mario Agio, Emiliano Descrovi, Ulrich Jonas

**Affiliations:** 1 Department Chemistry—Biology, University of Siegen, Adolf-Reichwein-Strasse 2, 57076 Siegen, Germany; 2 Department Physics, University of Siegen, Walter-Flex-Str. 3, 57072 Siegen, Germany; 3 National Institute of Optics (INO), National Research Council (CNR), Via Nello Carrara 1, 50019 Sesto Fiorentino, Italy; 4 Department of Applied Science of Technology (DISAT), Politecnico di Torino, Corso Duca degli Abruzzi 24, 10129 Torino, Italy

In the original publication [1], there was a mistake in Figure 2 as published. Parts (c) and (d) were mistakenly exchanged with parts (e) and (f), respectively. Thus, the figure caption did not match the content. The corrected Figure 2 appears below. The authors state that the scientific conclusions are unaffected. This correction was approved by the Academic Editor. The original publication has also been updated.

## Figures and Tables

**Figure 2:**
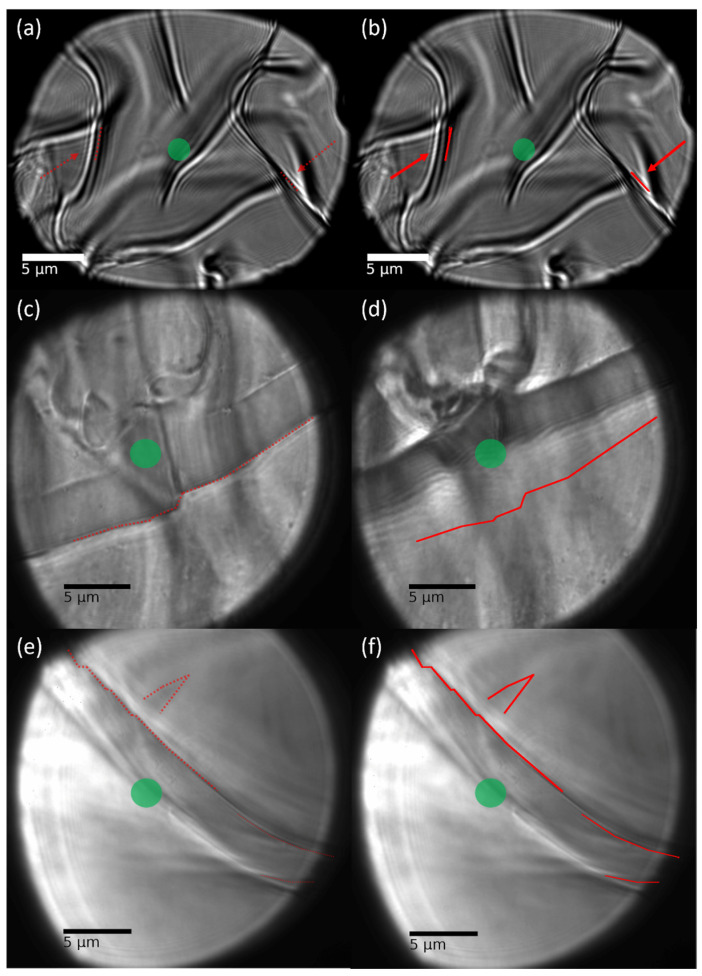
Light microscopy images of pristine (left panels) and photoactuated regions (right panels) of gel layers prepared from (**a**,**b**) poly(HEAm_96.5%_-co-o-MREAm_2.5%_-co-BPAAm_1%_) P_NR_, swollen in water, (**c**,**d**) poly(NipAAm_86.5%_-HEAm_10%_-co-o-MREAm_2.5%_-co-BPAAm_1%_) P_22°C_, swollen in water, (**e**,**f**) the same polymer in isopropanol. Photoactuation was performed with a laser at λ = 532 nm and 2600 µW (irradiation region marked by a green spot). Red lines and arrows indicate notable gel features that were photoactuated. Videos of the photomotion are provided in the download section of the supporting information.
