# Supplementary material for: Photomotion of Hydrogels with Covalently Attached Azo Dye Moieties—Thermoresponsive and Non-Thermoresponsive Gels
Source: Gels. 2022 Aug 28;8(9):541. doi: 10.3390/gels8090541 (PMC9498539; doi:10.3390/gels8090541)
Supplement: Supplementary file 1 [file gels-08-00541-s001.zip › Photomotion of Hydrogels with Azobenzene Moieties ESI Gels rev1.pdf]

Electronic Supporting Information (ESI)

Photomotion of Hydrogels with Covalently Attached Azobenzene Moieties – Thermoresponsive and Non-Thermoresponsive Gels

Thorben G. Jaik[1], Assegid M. Flatae[2], Navid Soltani[2], Philipp Reuschel[2], Mario Agio[2,3], Emiliano Descrovi[4], Ulrich Jonas[1]*

[1] Department Chemistry - Biology, University of Siegen, Adolf-Reichwein-Strasse 2, 57076, Siegen, Germany

[2] Department Physics, University of Siegen, Walter-Flex-Str. 3, 57072 Siegen, Germany

[3] National Institute of Optics (INO), National Research Council (CNR), via Nello Carrara 1, 50019 Sesto Fiorentino, Italy

[4] Department of Applied Science of Technology (DISAT), Politecnico di Torino, Corso Duca degli Abruzzi 24, 10129 Torino, Italy

*Corresponding author: Prof. Dr. Ulrich Jonas, jonas@chemie.uni-siegen.de

This document contains experimental details like syntheses and sample preparations, additional optical analysis data, and light microscopy images of polymer films with or without irradiation.

Experimentals

Materials and equipment:

All solvents used were of Milli-Q®, spectroscopic or HPLC-grade. 1,4-Dioxane was dried and distilled over sodium. Absolute ethanol was purchased from VWR Chemicals. *N*-hydroxyethyl acrylamide (97%, Sigma Aldrich) was used as received. *N*-Isopropylacrylamide was recrystallised from n-hexane. Azobisisobutyronitrile was recrystallised from methanol. 4-Benzophenoneacrylamide was synthesised according to literature [43]. The synthesis of the *N*-hydroxyethyl acrylamide ester of *o*-methyl red was described in a previous publication [44]. 4-(3-Triethoxysilyl)-

propoxybenzophenone was synthesised by Mr. Daniel John according to literature [45].
UV-vis measurements were performed on a Thermo Scientific™ Evolution™ 220 UV-Vis-spectrophotometer. If not stated otherwise, the measurements were done with 100 nm/min and a resolution of 1 nm. Cloud point measurements were performed with a heating/cooling rate of 1 K/min at mass per volume concentrations of 1%.
NMR measurements were performed on either a Bruker AV 400 or a Jeol EZC 500.
GPC/SEC was measured on a PSS GRAM linM column (Polymer Standards Service GmbH, Mainz, Germany) in dimethylacetamide with LiBr (0.1 g/L) at 60 °C with PMMA-standards as reference.

Free radical copolymerisations:
The different copolymers were obtained by radical polymerisation. The monomers (0.6 mol L^{-1}) and azobisisobutyronitrile were dissolved in 1,4-dioxane or methanol. The solutions were purged with nitrogen for 30 minutes and heated in an oil bath at 60 °C in methanol or 1,4-dioxane for 24 to 48 hours. The polymers were then precipitated up to three times in a non-solvent. The details are summarised in Table S 1.

P_{NR} (poly(HEAm-co-o-MREAm-co-BPAAm)): δH(500 MHz, MeOD) 8.5-6.5 (br, aromatic H), 4.57 (COOCH_2), 4.38 (Ar-CONHCH_2), 3.66 (CH_2CH_2OH), 3.51-3.12 (CONHCH_2), 2.4-1.25 (backbone)
$P_{22°C}$ (poly(NiPAAm-co-HEAm-co-o-MREAm-co-BPAAm)): δH(400 MHz, MeOD) 8.0-6.75 (br, aromatic H), 4.38 (Ar-COOCH_2), 3.96 (NHC*H*(CH_3)$_2$), 3.65 (CH_2CH_2OH), 3.12 (N(Me)$_2$), 2.3-1.25 (backbone), 1.16 (NHCH(CH_3)$_2$)
$P_{35°C}$ (poly(NiPAAm-co-HEAm-co-o-MREAm-co-BPAAm)): δH(500 MHz, D_2O) 8.1-6.75 (br, aromatic H), 3.90 (NHC*H*(CH_3)$_2$), 3.69 (CH_2CH_2OH), 3.37 (CONHCH_2), 2.5-1.25 (backbone), 1.16 (NHCH(CH_3)$_2$)
$P_{45°C}$ (poly(NiPAAm-co-HEAm-co-o-MREAm-co-BPAAm)): δH(400 MHz, D_2O) 8.0-6.75 (br, aromatic H), 3.88 (NHC*H*(CH_3)$_2$), 3.66 (CH_2CH_2OH), 3.33 (CONHCH_2), 2.3-1.25 (backbone), 1.13 (NHCH(CH_3)$_2$)

Table S 1: Feed composition in mol% and reaction parameters for the different free radical copolymersations.

Polymer	NiPAAm [%]	HEAm [%]	o-MREAm [%]	BPAAm [%]	AIBN [%]	T [°C]	Time [h]	Solvent	Non-solvent
**NR**	0	96.5	2.5	1	2	60	24	Methanol	Ethyl acetate
**22 °C**	87.2	9.2	2.6	1	2	60	26	1,4-Dioxane	Diethylether
**35 °C**	55.9	40.4	2.7	1	2	60	24	Methanol	Diethylether
**45 °C**	27.7	67.3	2.5	2.5	2	60	48	Methanol	Diethylether

Table S 2: Copolymer characteristics as determined by UV-vis spectroscopy and GPC. The built-in ratios for o-MREAm and BPAAm are given in weight percentages and the percentage of the monomer built-in compared to the feed composition. The HEAm:NiPAAm ratio was determined by ^{1}H NMR spectroscopy.

Polymer	o-MREAm [w%] Built-in to feed (%)	BPAAm [w%] Built-in to feed (%)	HEAm:NipAAm ratio; Built-in to feed (mol%)	M_n [10^3 Da]	M_w [10^3 Da]	*Đ*	Yield [%]
**NR**	4.5 (65%)	1.9 (94%)	-	17.4	37.7	2.17	79
**22 °C**	5.2 (67%)	2.1 (96%)	0.14 (133%)	22.1	51.9	2.35	81
**35 °C**	5.5 (68%)	2.4 (120%)	0.83 (115%)	13.6	26.3	1.94	69
**45 °C**	4.4 (73%)	4.2 (84%)	2.53 (104%)	12.6	29.0	2.30	85

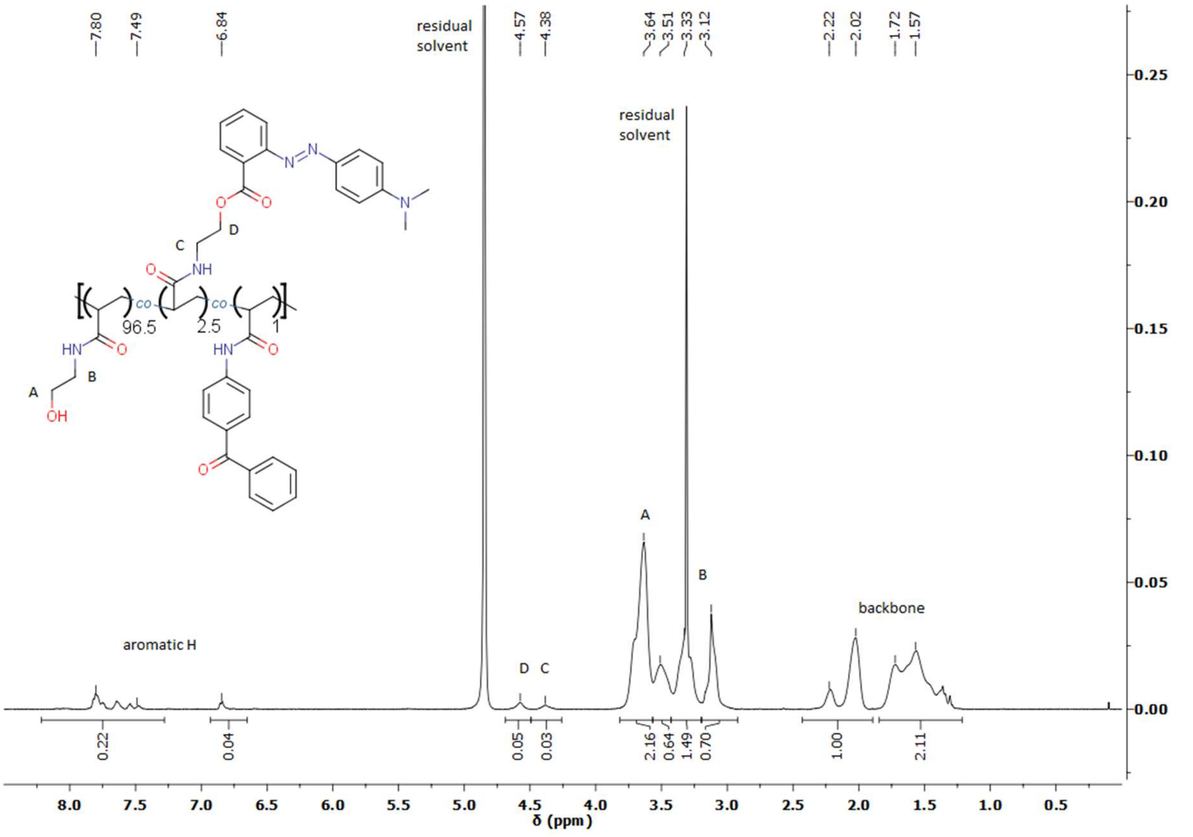

Figure S 1: ^{1}H NMR spectrum (500 MHz) in MeOD of a poly(HEAm-co-o-MREAm-co-BPAAm) copolymer (P_{NR}) (feed: HEAm:o-MREAm:BPAAm 96.5:2.5:1).

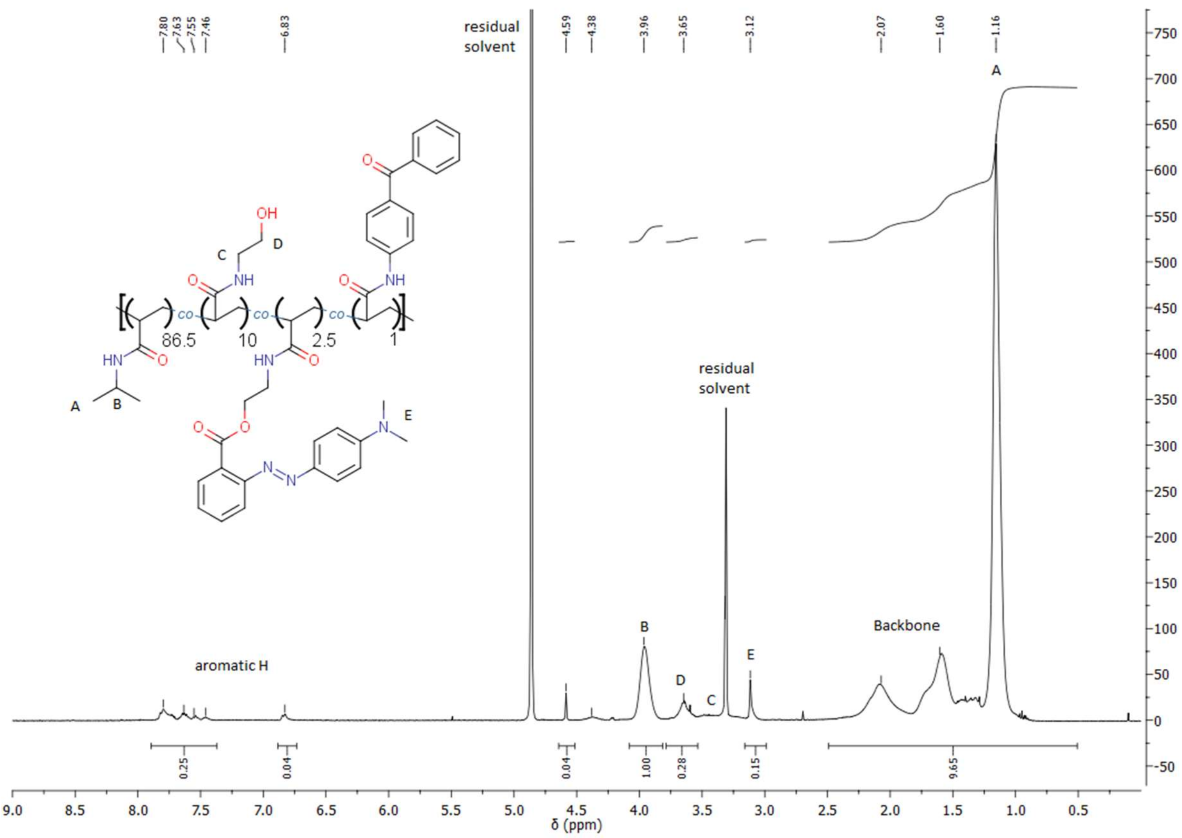

Figure S 2: ^{1}H NMR spectrum (400 MHz) in MeOD of a poly(NiPAAm-co-HEAm-co-o-MREAm-co-BPAAm) copolymer ($P_{22°C}$) (feed: NiPAAm:HEAm:o-MREAm:BPAAm 87.2:9.2:2.6:1).

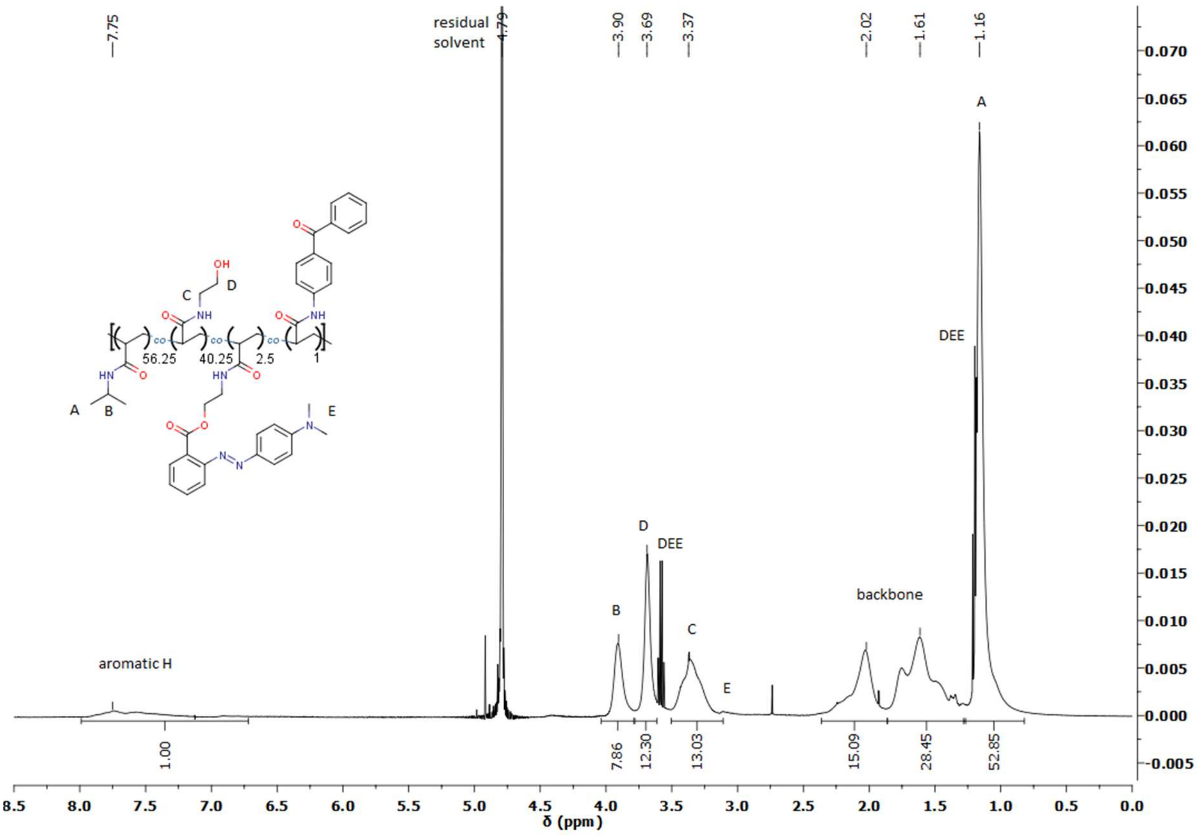

Figure S 3: ^{1}H NMR spectrum (500 MHz) in D_2O of a poly(NiPAAm-co-HEAm-co-o-MREAm-co-BPAAm) copolymer ($P_{35°C}$) (feed: NiPAAm:HEAm:o-MREAm:BPAAm 55.9:40.4:2.7:1).

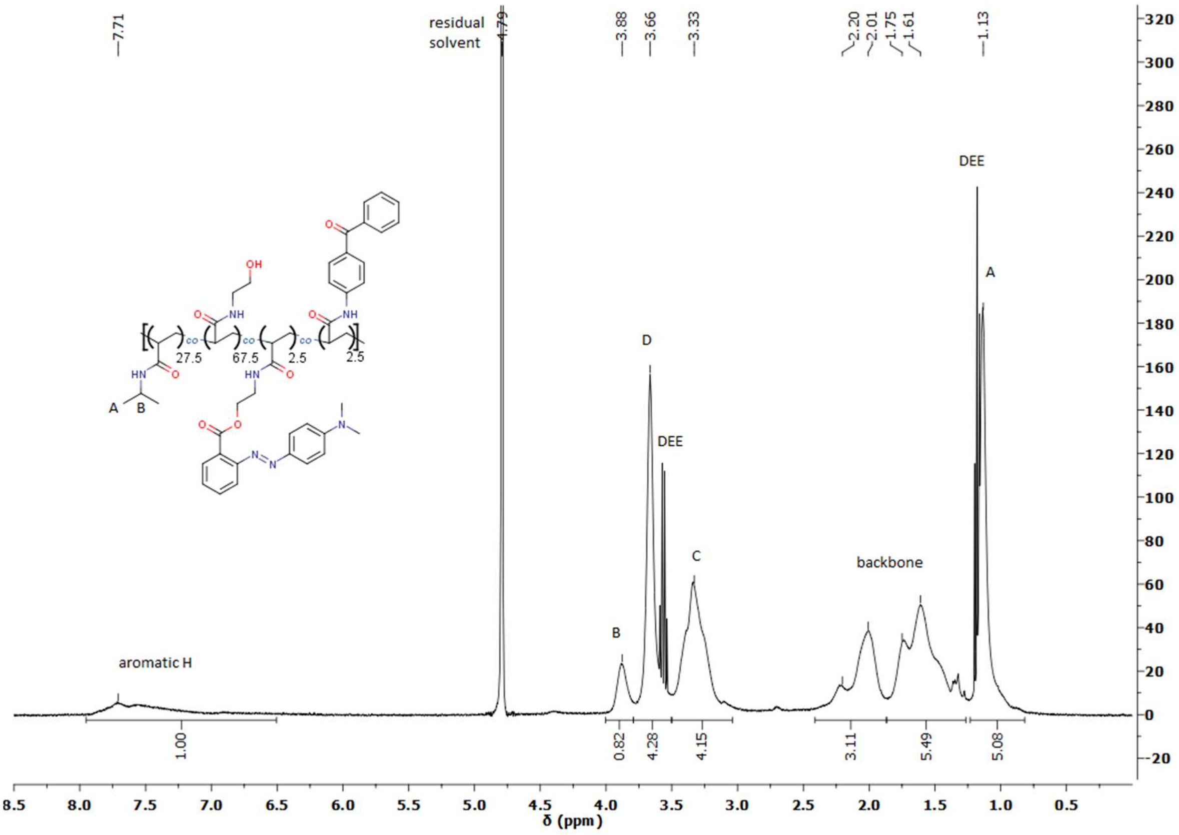

Figure S 4: ^{1}H NMR spectrum (500 MHz) in D_2O of poly(NiPAAm-co-HEAm-co-o-MREAm-co-BPAAm) copolymer ($P_{45°C}$) (feed: NiPAAm:HEAm:o-MREAm:BPAAm 27.7:67.3:2.5:2.5).

Film preparation:

The glass slides used in photoactuation experiments were cleaned with fresh Carothers' acid (sulfuric acid:hydrogen peroxide, 3:1) and rinsed thoroughly with water. The slides were dried under a nitrogen stream. They were submerged in an ethanolic solution of benzophenone silane (1 mmol L^{-1}) for 24 hours before they were rinsed thrice with absolute ethanol and finally dried under a nitrogen stream.

Polymers were drop-casted on the glass slides from MeOH:H_2O (4:1, P_{NR}, $P_{35°C}$, $P_{45°C}$) or aqueous ($P_{22°C}$) solution (2.5 w%, 25 µL). Photocrosslinking was performed at 302 nm with an energy of 20.3 J cm^{-2}. All films were washed with water before further experiments.

Light Microscopy and Photoactuation Set-Up:

The laser used in the experiments is a Class IIIb CW laser with a wavelength of 532 nm and 10 mW power, model MGLIII532, by Changchun New Industries Optoelectronics Tech Co., Ltd., People's Republic of China.

The camera used for imaging is a Zyla sCMOS by Andor Oxford Instruments, UK, with the matching Andor SOLIS software.

Laser power was determined before the objective with a PM100D power meter with a S130c 400 nm - 1100 nm probe by ThorLabs GmbH, Germany.

The objective used is an EC Epiplan-APOCHROMAT 50x/0.95 DIC by ZEISS, Germany.

A 542 nm long-pass filter by ThorLabs GmbH, Germany, was places in front of the camera.

The set-up is sketched in Figure 1 of the main article.

Optical Data and Light Microscopy Images:

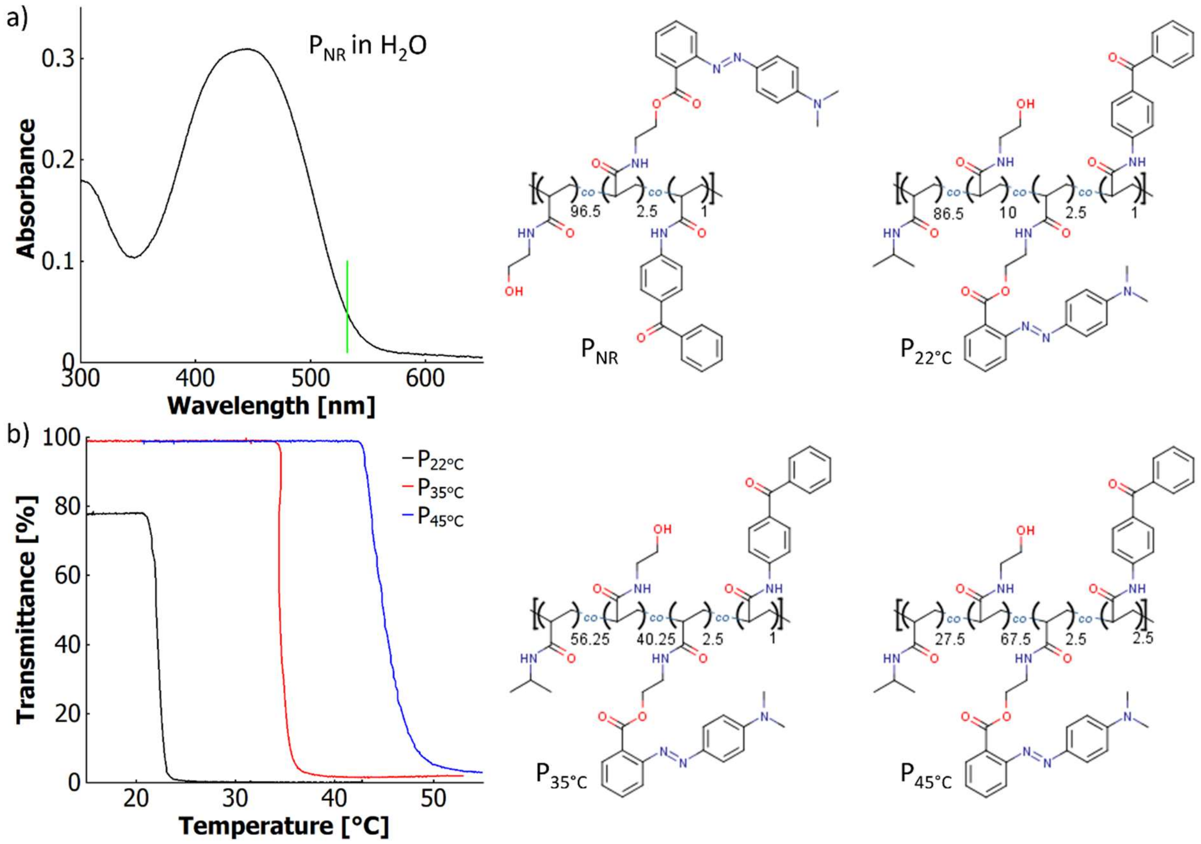

Figure S 5: a) UV-vis spectrum of a photocrosslinked film of poly(HEAm$_{96.5\%}$-co-o-MREAm$_{2.5\%}$-co-BPAAm$_{1\%}$) P$_{NR}$ swollen in water at 26 °C, with the wavelength of the green laser used in this marked with a green vertical line at 532 nm. b) Turbidity measurements at 780 nm of aqueous 1 w% solutions of poly(NipAAm$_{86.5\%}$-HEAm$_{10\%}$-co-o-MREAm$_{2.5\%}$-co-BPAAm$_{1\%}$) P$_{22°C}$, poly(NipAAm$_{56.25\%}$-HEAm$_{40.25\%}$-co-o-MREAm$_{2.5\%}$-co-BPAAm$_{1\%}$) P$_{35°C}$, and poly(NipAAm$_{27.5\%}$-HEAm$_{67.5\%}$-co-o-MREAm$_{2.5\%}$-co-BPAAm$_{2.5\%}$) P$_{45°C}$. On the right: structures of the copolymers used in this study with the feed ratios of monomers in the synthesis.

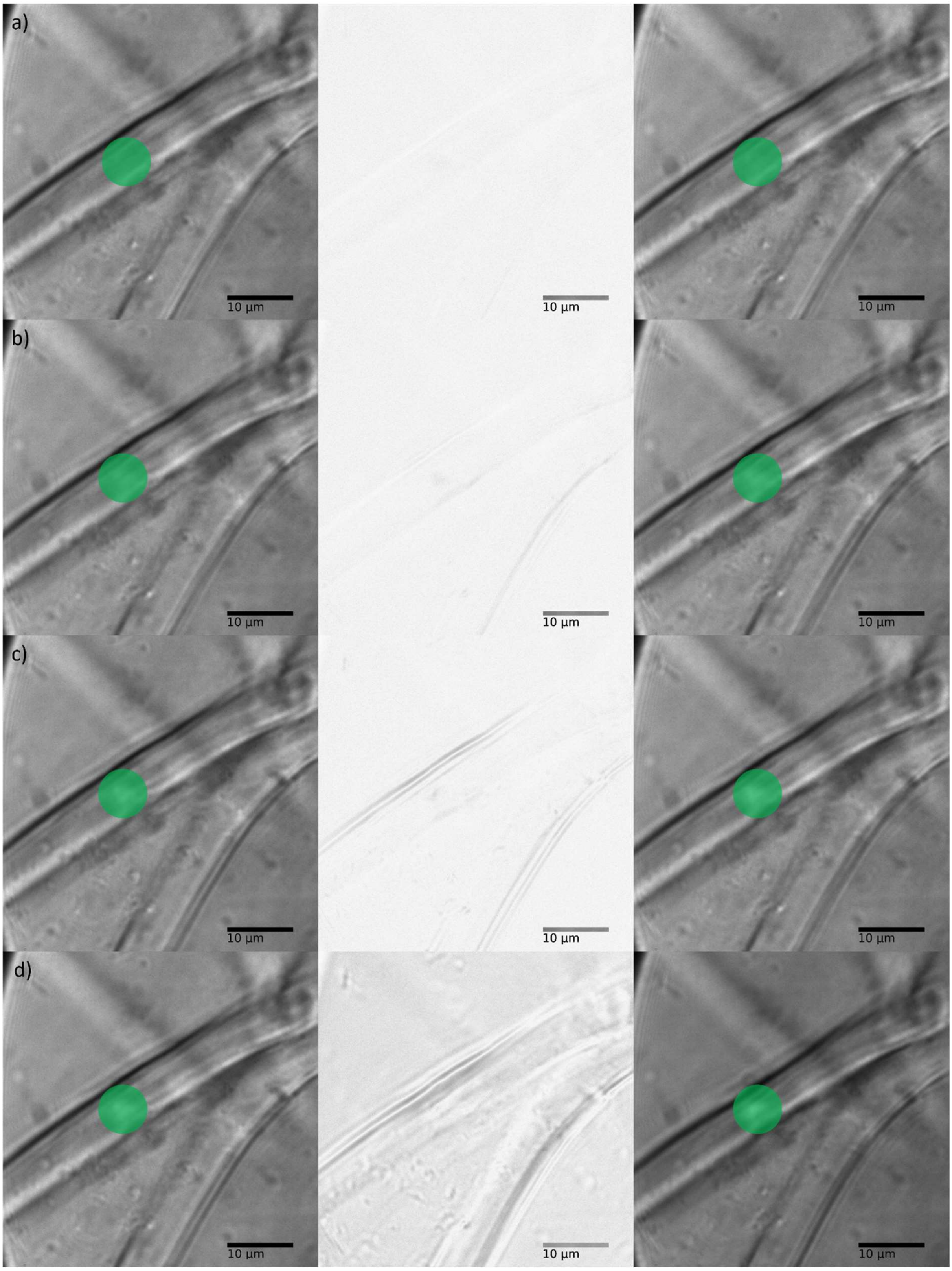

Figure S 6: A series of light microscopy images of a hydrogel of poly(NipAAm$_{86.5\%}$-HEAm$_{10\%}$-co-o-MREAm$_{2.5\%}$-co-BPAAm$_{1\%}$) P$_{22°C}$ showing left: non-irradiated gel with accumulative irreversible change of irradiations at previous stages of power; middle: difference pictures between the non-irradiated and the irradiated gel with inverted colour scheme (laser at λ=532 nm); right: irradiated gel after 3 s at a) 85 µW, b) 376 µW, c) 1210 µW, and d) 3750 µW. The green shaded area shows the approximate dimensions of the laser spot.

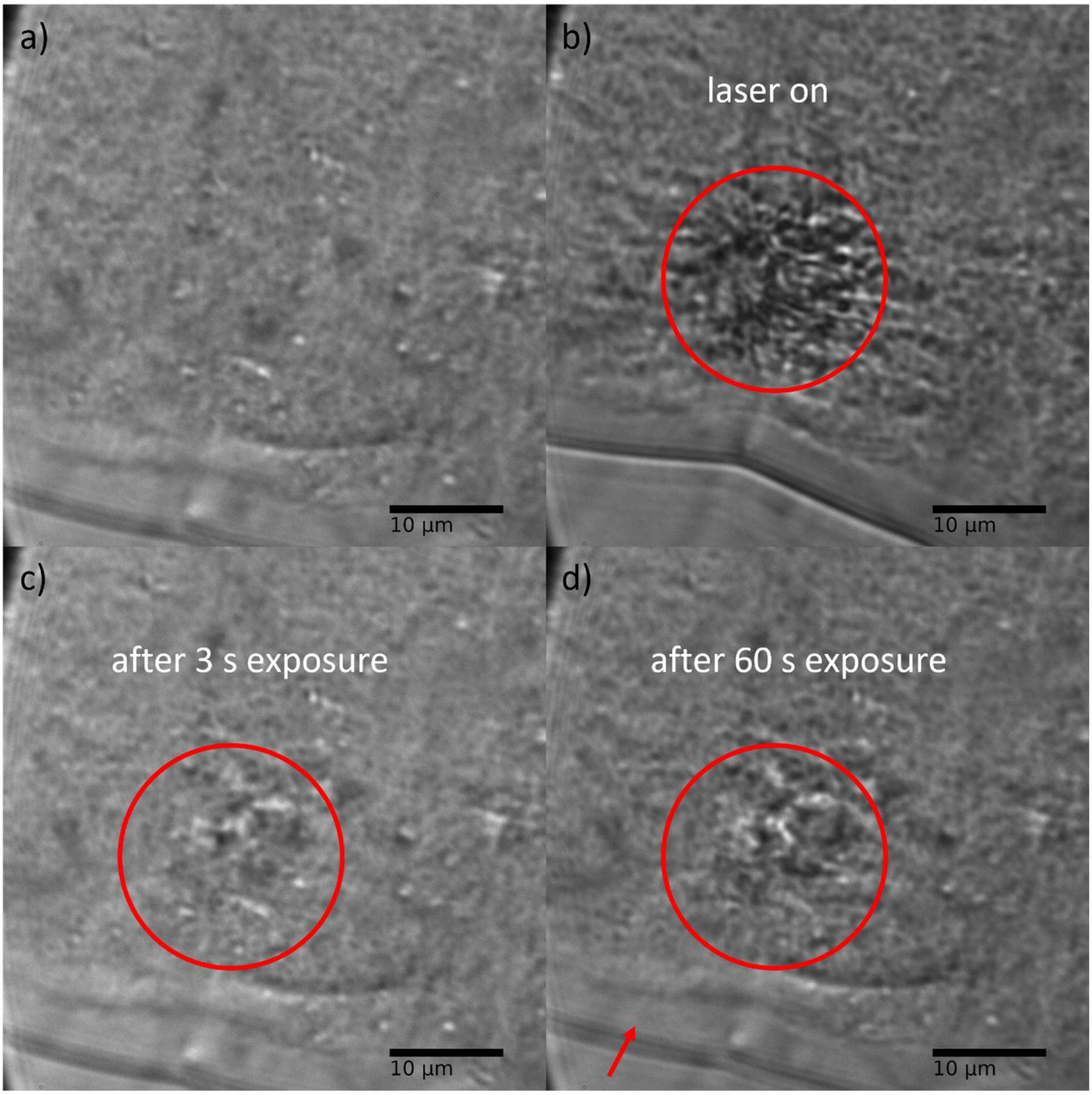

Figure S 7: Light microscopy images of a hydrogel of poly(NipAAm$_{86.5\%}$-HEAm$_{10\%}$-co-o-MREAm$_{2.5\%}$-co-BPAAm$_{1\%}$) P$_{22°C}$ showing a) the pristine hydrogel, b) the hydrogel when irradiated with a laser (λ=532 nm, 3750 µW), c) after 3 s of exposure, and d) after 60 s of exposure.

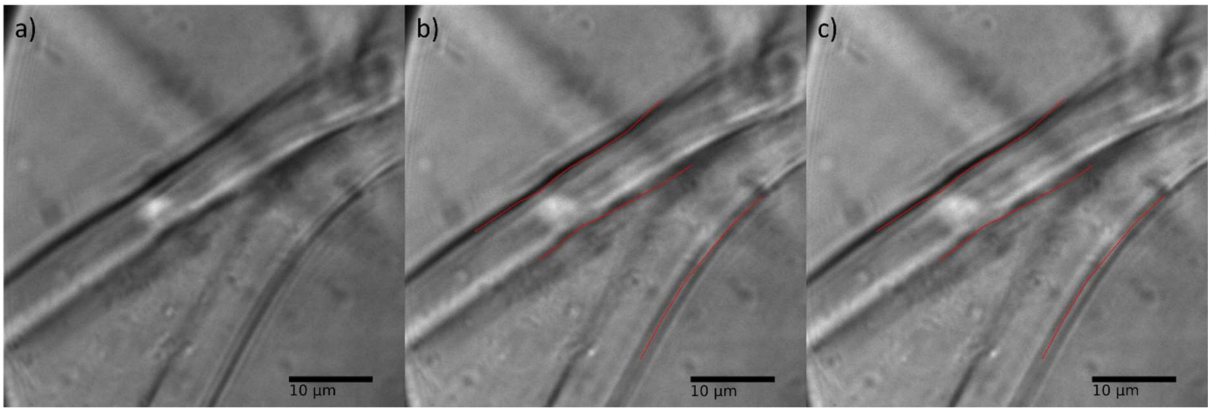

Figure S 8: Light microscopy images of a hydrogel of poly(NipAAm$_{86.5\%}$-HEAm$_{10\%}$-co-o-MREAm$_{2.5\%}$-co-BPAAm$_{1\%}$) P$_{22°C}$ irradiated with laser (λ=532 nm) at 3750 µW a) for the first time, b) after cycling five times between 3 s irradiation intervals and relaxation, c) after cycling twenty times between 3 s irradiation intervals and relaxation. The red lines in b) and c) show the positions of the notable dark features in a).

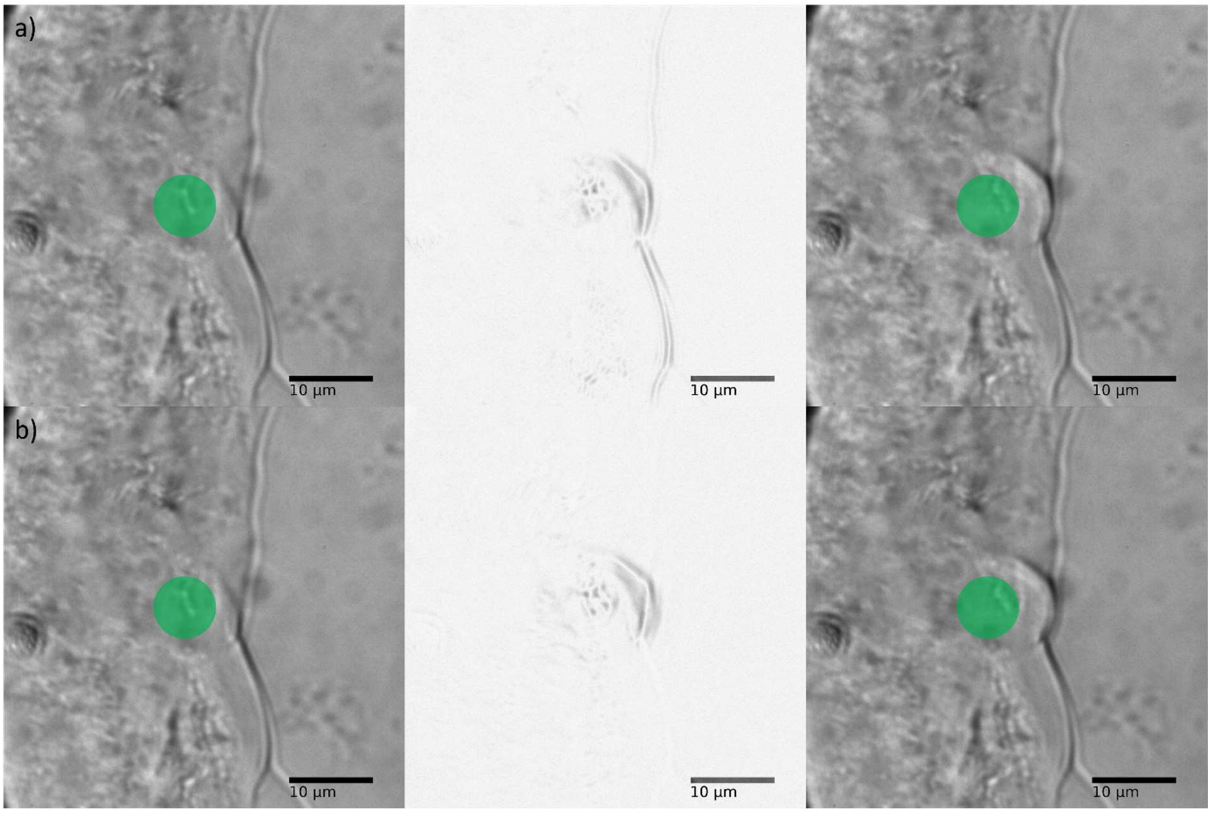

Figure S 9: Light microscopy images of a hydrogel of poly(NipAAm$_{56.25\%}$-HEAm$_{40.25\%}$-co-o-MREAm$_{2.5\%}$-co-BPAAm$_{1\%}$) P$_{35°C}$ showing a) left to right: the pristine gel, a difference picture between the non-irradiated and the irradiated gel, the irradiated gel (laser at λ=532 nm, 3750 µW), and b) left to right: the pristine gel, a difference picture between the non-irradiated gel before and after exposure to laser light, the gel after exposure to laser light. The green shaded area shows the approximate dimensions of the laser spot. The difference pictures are shown with inverted colour scheme.

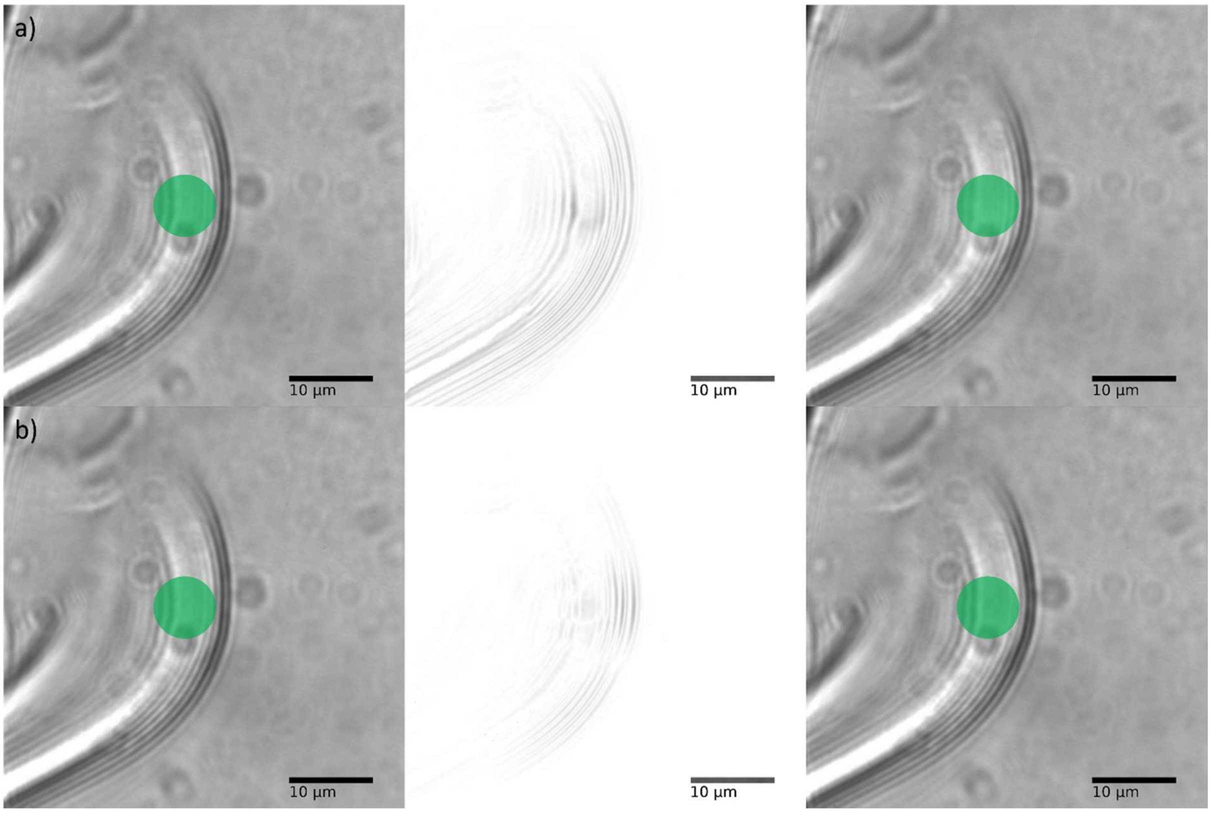

Figure S 10: Light microscopy images of a hydrogel of poly(NipAAm$_{27.5\%}$-HEAm$_{67.5\%}$-co-o-MREAm$_{2.5\%}$-co-BPAAm$_{2.5\%}$) P$_{45°C}$ showing a) left to right: the pristine gel, a difference picture between the non-irradiated and the irradiated gel, the irradiated gel (laser at λ=532 nm, 3750 µW), and b) left to right: the pristine gel, a difference picture between the non-irradiated gel before and after exposure to laser light, the gel after exposure to laser light. The green shaded area shows the approximate dimensions of the laser spot. The difference pictures are shown with inverted colour scheme.

References

43. Jia, J.; Sarker, M.; Steinmetz, M.G.; Shukla, R.; Rathore, R. Photochemical elimination of leaving groups from zwitterionic intermediates generated via electrocyclic ring closure of alpha,beta-unsaturated anilides. *J. Org. Chem.* **2008**, *73*, 8867–8879, doi:10.1021/jo8017445.
44. Jaik, T.G.; Ciubini, B.; Frascella, F.; Jonas, U. Thermal Response and Thermochromism of Methyl Red-Based Copolymer Systems - Coupled Responsiveness in Critical Solution Behaviour and Optical Absorption Properties. *Polym. Chem.* **2022**, *13*, 1186–1214, doi:10.1039/D1PY01361K.
45. Gianneli, M.; Roskamp, R.F.; Jonas, U.; Loppinet, B.; Fytas, G.; Knoll, W. Dynamics of swollen gel layers anchored to solid surfaces. *Soft Matter* **2008**, *4*, 1443–1447, doi:10.1039/b801468j.
